# Atherosclerosis in Patients with Congenital Hemophilia: A Focus on Peripheral Artery Disease

**DOI:** 10.3390/life13112221

**Published:** 2023-11-18

**Authors:** Minerva Codruta Badescu, Oana Viola Badulescu, Alexandru Dan Costache, Ovidiu Mitu, Vasile Valeriu Lupu, Bianca-Ana Dmour, Ancuta Lupu, Liliana Georgeta Foia, Irina-Iuliana Costache, Ciprian Rezus

**Affiliations:** 1 Department of Internal Medicine, “Grigore T. Popa” University of Medicine and Pharmacy, 16 University Street, 700115 Iasi, Romania; minerva.badescu@umfiasi.ro (M.C.B.); ovidiu.mitu@umfiasi.ro (O.M.); gherasimbianca93@gmail.com (B.-A.D.); irina.costache@umfiasi.ro (I.-I.C.); ciprian.rezus@umfiasi.ro (C.R.); 2 III Internal Medicine Clinic, “St. Spiridon” County Emergency Clinical Hospital, 1 Independence Boulevard, 700111 Iasi, Romania; 3 Department of Pathophysiology, “Grigore T. Popa” University of Medicine and Pharmacy, 16 University Street, 700115 Iasi, Romania; 4 Hematology Clinic, “St. Spiridon” County Emergency Clinical Hospital, 1 Independence Boulevard, 700111 Iasi, Romania; 5 Cardiovascular Rehabilitation Clinic, Clinical Rehabilitation Hospital, 700661 Iasi, Romania; 6 Cardiology Clinic, “St. Spiridon” County Emergency Clinical Hospital, 1 Independence Boulevard, 700111 Iasi, Romania; 7 Pediatrics Department, Faculty of Medicine, “Grigore T. Popa” University of Medicine and Pharmacy, 16 University Street, 700115 Iasi, Romaniaanca_ign@yahoo.com (A.L.); 8 Department of Biochemistry, “Grigore T. Popa” University of Medicine and Pharmacy, 16 University Street, 700115 Iasi, Romania; lilifoia@yahoo.co.uk

**Keywords:** atherosclerosis, peripheral artery disease, hemophilia, endothelium, biomarkers, antiplatelet drugs

## Abstract

Advances in the treatment of hemophilia have increased the life expectancy of this population and we are currently facing diseases associated with aging, including cardiovascular ones. Coronary atherosclerosis, with acute myocardial infarction as the most severe form of manifestation, has been recognized as part of the comorbidities of hemophiliacs. However, little is known about peripheral artery disease. Available data show that hemophiliacs have cardiovascular risk factors and atherosclerosis similar to the general population. Impaired thrombus formation and phenotype of atheroma plaque rather than the burden of atherosclerosis explains their lower cardiovascular mortality. Since the effect of traditional cardiovascular risk factors overpowers that of decreased coagulability and promotes the onset and progression of atherosclerotic lesions, screening for traditional cardiovascular risk factors and peripheral artery disease should be integrated into standard hemophilia care. There is evidence that invasive treatments and long-term antithrombotic therapy are generally safe, provided that coagulation factor levels are taken into account and replacement therapy is given when necessary.

## 1. Introduction

Congenital hemophilia is the most frequent severe inherited bleeding disorder [1]. Because it has a recessive X-linked inheritance pattern, men develop disease while women are carriers. There are three types of congenital hemophilia, depending on the deficient coagulation factor. Hemophilia A (HA) is the consequence of coagulation factor VIII (FVIII) deficiency. Its prevalence is 1 in 5000 live male births, being the most widespread form (80–85% of all hemophiliacs). Hemophilia B (HB) is the result of coagulation factor IX (FIX) deficiency and has a lower prevalence, of 1 in 30,000 live male births. Deficiency of coagulation factor XI is hemophilia C and is very rare. Depending on the coagulation factor activity present in the blood, hemophilia is mild (>5% to <40%), moderate (1–5%) or severe (<1%) [2].

Due to advances in the treatment of hemophilia, hemophiliacs are now reaching an old age similar to that of the general population. With aging, accumulation of traditional cardiovascular risk factors—hypertension, diabetes mellitus, obesity and dyslipidemia—were noted in individuals with hemophilia [3]. Fear of trauma with bleeding and hemophilic arthropathy, which causes pain and restriction of movement in large joints of lower limbs, are the main determinants of sedentarism. Lack of physical activity leads to being overweight and to obesity, which further constrains mobility. Estimates from Europe and North America show that one in three hemophiliacs is overweight or obese [4]. When restricted to adults, the prevalence of overweight/obesity is higher (43.3%), with significant differences between Europe (49.1%) and North America (38.5%). Although overweight/obesity was not a risk factor for cardiovascular events in North American hemophiliacs [5], body mass index (BMI) was significantly associated with an increased risk for hypertension in European hemophiliacs [6]. Prevalence of diabetes mellitus and smoking is not different between hemophiliacs and the general population. Prevalence of hyperlipidemia is low in hemophiliacs, at 0.4%, most likely due to the coexistence of liver dysfunction induced by the hepatitis C virus [3]. It must be emphasized that arterial hypertension is more prevalent in hemophiliacs than in the general population [7].

Patients with hemophilia have low cardiovascular mortality and there were intense debates about whether it is due to decreased thrombin generation that results in inhibition of thrombus formation or whether they develop less atherosclerosis [8]. Overt coronary artery disease (CAD) is present in patients with hemophilia, and its prevalence increases with age, from 0.05% in those under 30 to 15.2% in those 60 years or older [9]. Although its prevalence is lower than in the general population, hospital discharge records showed that the rate of CAD was half in hemophiliacs than in non-hemophilic men for ages 45–64 and only 30% lower in those 65 years or older. Therefore, in hemophiliacs, there is an increase in the prevalence of atherosclerotic coronary artery disease with aging.

Peripheral artery disease (PAD) shares the same risk factors and substrate—atherosclerosis—with CAD. Moreover, multisite artery disease increases the overall risk of cardiovascular events and it is associated with the worst clinical outcomes [10]. In a broad concept, PAD includes all arterial diseases other than coronary arteries and aorta. However, since intracranial arterial diseases are often managed by other specialists than cardiologists and vascular surgeons, the term PAD used in our paper parallels that of the 2017 European Society of Cardiology Guidelines on the Diagnosis and Treatment of Peripheral Arterial Diseases [10]. Therefore, it includes atherosclerotic disease of extracranial carotid and vertebral, mesenteric, renal, upper and lower extremity arteries.

While there is a large amount of data on subclinical and clinical overt atherosclerosis in the general population, there are extremely limited published results in hemophiliacs. Our narrative review aims to provide a comprehensive analysis of available literature and an up-to-date overview of PAD in hemophiliacs. Firstly, we aimed to investigate whether there is enough evidence to support screening for subclinical atherosclerosis in hemophiliacs. Secondly, we tried to find out if prevention/treatment measures should be instituted and what would these be, considering that hemophiliacs are a population with high bleeding risk and that in the general population, antiplatelet therapy is recommended to all patients with established atherosclerotic cardiovascular disease (CVD) and in some patients at high or very high CVD risk [11]. As far as we know, our paper is the first synthesis of PAD in hemophiliacs.

## 2. Subclinical Atherosclerosis

Intima-media thickness (IMT) is a parameter assessed by vascular ultrasound and can be used as a measure of subclinical atherosclerosis (Table 1). Adding common carotid IMT measurement to traditional cardiovascular risk scores allows a slightly better estimation of 10-year individual risk of stroke and myocardial infarction in individuals framed by Framingham Risk Score in the intermediate-risk group [12]. As a consequence of reclassification, some patients will be placed in the high-risk category and will have the advantage of early implementation of adequate management. IMT measurement in the common femoral artery is more sensitive than that in the carotid artery to identify early atherosclerotic changes. Intima-media thickening in femoral arteries occurs earlier and reflects better than in the carotid extent of systemic atherosclerosis [13].

Furthermore, vascular ultrasound is useful for the identification and characterization of atherosclerotic plaques and allows quantification of the severity of stenosis they produce. For this assessment, B-Mode ultrasound is used in combination with spectral and color Doppler.

### 2.1. Subclinical Atherosclerosis in Carotid Arteries

The first carotid ultrasonography-based study showed a lower prevalence of atherosclerotic plaques and less severe stenosis in patients with coagulopathy than in controls, even if traditional cardiovascular risk factors were present in cases and absent in controls [14]. Prevalence of plaques was 13.1% in cases and 27.2% in controls. This difference increased significantly with advanced age, from 5.3% vs. 12.5% in patients younger than 60 years to 19.7% vs. 62.5% in those older than 60 years. Among patients with coagulopathy, those with more severe disease had fewer atheroma plaques. Furthermore, no patient with coagulopathy had severe carotid stenosis. These results are consistent with IMT values from a subsequent study [15]. In the presence of a similar amount of traditional cardiovascular risk factors, mean IMT was significantly lower in hemophiliacs than in controls.

Other studies found no difference in IMT between patients with coagulopathy and controls [16,17]. No clinically relevant effect of hypocoagulability on atherogenesis was found in a northern European study that compared coagulopathic patients with controls with a similar burden of traditional cardiovascular risk factors [16]. Sartori et al. showed that IMT in hemophiliacs was similar to controls, even though most participants had at least one traditional cardiovascular risk factor, and nearly half had two or more [17]. Another study confirmed that mean IMT was not different in hemophiliacs from controls, even when comparison was restricted to those with obesity or with severe and moderate hemophilia [18]. The only significant difference in mean IMT was between obese and non-obese subjects. Carotid plaques were present in 33% of patients and 25% of controls (*p* = 0.25) and their prevalence did not correlate with severity of hemophilia or with obesity. As expected, mean IMT increased progressively with aging, similarly in hemophiliacs and controls.

A large analysis of patients with coagulopathy found a mean IMT of the carotid artery of 0.75 mm in cases and of 0.74 mm in age- and sex-matched healthy controls [8]. A study that included only hemophiliacs found a mean IMT of 0.80 mm, which was within age-specific local reference values [19]. There was no variability in IMT with severity (*p* = 0.81) or type of hemophilia (*p* = 0.78). Mean values for IMT in patients with mild, moderate and severe disease were 0.76 mm, 0.77 mm and 0.74 mm, respectively. However, there was a statistically significant difference in median IMT between hemophiliacs with and without previous major adverse cardiovascular events (1.09 mm vs. 0.76 mm, *p* < 0.0010).

**Table 1:** Major studies assessing peripheral artery disease.

Author, Year	Country	No. of Patients (Cases)	Mean/Median Age (Years)	HA	HB	Mild	Moderate	Severe	Control Group		IMT and/or FMD	Study Conclusions
									No	Selection Criteria		
Bilora et al., 1999 [14]	Italy	76 ^a^	58.2	64	0	NR	NR	NR	77	No atherosclerosis risk factors	IMTc	Fewer plaques, less severe stenosis in cases than controls; Fewer plaques in cases with more severe disease.
Sramek et al., 2001 [16]	Netherlands	76 ^a^	48.8	52	7	34	5	20	142	Healthymen	IMTc IMTf	IMTc in cases similar to controls; IMTf slightly lower in cases than controls.
Bilora et al., 2001 [20]	Italy	40 ^a^	48.3	25	0	NR	NR	NR	40	Matched for age, sex, atherosclerosis risk factors	NA	Fewer plaques, less severe stenosis in cases than controls; Fewer plaques in cases with more severe disease.
Bilora et al., 2006 [15]	Italy	50	41.72	50	0	0	12	38	50	Matched for age, sex; free of symptomatic atherosclerosis	IMTc IMTb IMTa IMTf	Significantly fewer plaques, less severe stenosis in cases than controls.
Sartori et al., 2008 [17]	Italy	40	39.5 ^b^ 49.5 ^c^	38	2	16	24		40	Matched for age, sex, smoking habit, BMI, hypertension, diabetes mellitus and dyslipidemia	IMTc FMD	Similar mean IMTc; Significantly impaired mean FMD in cases than controls.
Zwiers et al., 2012 [19]	Netherlands	69	52	51	18	34	8	27	-	Local reference values from healthy individuals	IMTc	Mean IMTc within age-specific reference values; Significant difference in median IMT between hemophiliacs with/without previous MACE.
Biere-Rafi et al., 2012 [18]	Netherlands	98 51 obese 47 non-obese	50	98	0	49	16	33	92	Matched for age, sex, BMI; 42 obese 50 non-obese	IMTc IMTf FMD	IMTc, IMTf, FMD and prevalence of atherosclerotic plaques in hemophiliacs were similar to controls; IMTc increased in obese as compared with non-obese subjects.

^a^ study included patients with von Willebrand disease; ^b^ hemophiliacs with moderate–severe disease; ^c^ hemophiliacs with mild disease; HA = hemophilia A; HB = hemophilia B; NR = not reported; NA = not assessed. IMTc = intima-media thickness of carotid artery; IMTb = intima-media thickness of brachial artery; IMTa = intima-media thickness of aorta; IMTf = intima-media thickness of femoral artery; FMD = flow-mediated dilation; BMI = body mass index; MACE = major adverse cardiovascular events.

### 2.2. Subclinical Atherosclerosis in Arteries of the Upper Limbs

IMT in the brachial artery was assessed in only one study [15]. Mean IMT was significantly lower in hemophiliacs than in controls, while the burden of traditional cardiovascular risk factors was similar.

### 2.3. Subclinical Atherosclerosis of the Arteries of the Lower Limbs

Plaques in the abdominal aorta and arteries of lower limbs were identified by ultrasound in three (7.5%) and five (12.5%) patients with coagulopathy, respectively, and in 11 (27.5%) and 17 (42.5%) controls, respectively [20]. Prevalence of traditional cardiovascular risk factors was similar between cases and controls. However, patients were relatively young, which could explain the generally low prevalence of atherosclerosis. Among patients with hemophilia, those with severe disease had the fewest atherosclerotic plaques in each of the two territories. In 11 hemophiliacs with mild disease, five plaques were identified in the arteries of lower limbs and two in the aorta, while in 14 hemophiliacs with moderate–severe disease, one plaque was identified in each territory. Another study found a mean IMT of the femoral artery of 0.75 mm in cases and 0.79 mm in age- and sex-matched healthy controls [8]. The study by Bilora et al. confirmed previous results [15]. Mean IMT measured in the abdominal aorta and femoral arteries was significantly lower in hemophiliacs than in controls, while the amount of traditional cardiovascular risk factors was similar.

In a northern European study, mean IMT was slightly lower in hemophiliacs than in controls, while the load of traditional cardiovascular risk factors was similar [16]. However, the difference had a low level of significance. Among hemophiliacs, patients with moderate and severe disease had an IMT of 0.74 mm, slightly lower than the 0.76 mm found in those with mild disease.

Only one study showed that mean IMT was not different in hemophiliacs from controls, even when comparison was restricted to those with obesity [18]. However, obese subjects tended to have a higher mean IMT than non-obese ones. Moreover, those with severe and moderate hemophilia tended to have a lower mean IMT than controls. As expected, mean IMT increased progressively with aging, similarly in hemophiliacs and controls.

## 3. Endothelial Dysfunction

Endothelial dysfunction is the first step in the development of atherosclerosis and an independent predictor for the occurrence of atherosclerotic events [21,22].

Macrovascular and microvascular endothelium-dependent function can be quantified by studying the phase of reactive hyperemia that follows an ischemic stimulus. Flow-mediated dilation (FMD) of a conduit artery, such as the brachial artery, is the most widely used method to assess macrovascular endothelium-dependent function [23]. FMD is a non-invasive physical technique based on ischemia-induced release of vasodilators, such as nitric oxide, from endothelium. Due to an increase in blood flow and therefore shear stress following an ischemic stimulus, arteries respond by dilation, a parameter that is easy to quantify. Microvascular endothelium-dependent function is approximated mathematically as the integrated total increase in flow velocity during the reactive hyperemia phase (hyperemic velocity time integral). The endothelium-dependent reactive hyperemia index (RHI) of digital arteries also assesses microvascular endothelium-dependent function. It reflects the change in arterial pulsatile volume after lifting occlusion of blood supply to the finger [24]. The higher the RHI value, the better the endothelial function and the lower the cardiovascular risk. Of note, microvascular endothelial dysfunction predicts cardiovascular risk better than macrovascular endothelial dysfunction [25].

Identification of circulating biomarkers of endothelial dysfunction emerges as an attractive strategy because it is free from shortcomings such as physiological variability and operator dependence.

### 3.1. Assessement of Reactive Hyperemia

The first study assessing endothelial function in hemophiliacs showed a significantly reduced endothelial function in cases compared to age-matched healthy controls, although IMT was similar in the two groups [17]. The severity of hemophilia did not influence the level of FMD impairment. Of note, most participants had at least one traditional cardiovascular risk factor, and nearly half of them had two or more.

Other studies did not confirm these results [18]. Biere-Rafi et al. found no difference in FMD between hemophiliacs and controls. Neither obesity nor hemophilia severity had any influence on FMD.

Macrovascular and microvascular endothelium-dependent function was assessed in 81 hemophiliacs and 243 healthy controls, mean age of 48 years in both groups [25]. On univariate analysis, macrovascular endothelium-dependent function was similar in cases and controls. However, on multivariable analysis, higher FMD was found in hemophiliacs. Microvascular endothelium-dependent function was significantly lower in hemophiliacs than in controls. Of note, neither baseline factor activity nor annualized factor utilization influenced macrovascular or microvascular endothelium-dependent function.

A pilot study of Böhmert et al. on endothelial function included 21 HA and 5 HB patients with moderate and severe disease [26]. The endothelium-dependent reactive hyperemia index of digital arteries was assessed. The RHI in hemophiliacs was 1.958 ± 0.358, a value that fell between that of the healthy control group (2.112 ± 0.601) and the group of patients with documented coronary artery disease (1.862 ± 0.553). None of the differences reached statistical significance. Considering that RHI values ≥ 2.1 indicate a low cardiovascular risk and 1.68–2 a moderate one and that hemophiliac values were lower than that of healthy controls, an increased cardiovascular risk could exist in patients with hemophilia.

### 3.2. Biomarkers of Endothelial Dysfunction

Increased or decreased blood concentrations of some substances synthesized within endothelial cells are established markers of endothelial injury and dysfunction [23]. Healthy endothelial cells synthesize tissue-type plasminogen activator (t-PA), a serine protease that converts inactive plasminogen to active enzyme plasmin, thereby activating intravascular fibrinolysis. t-PA has a continuous constitutive release that under stimulation is reinforced by a rapid release from the intracellular storage pool. Impaired t-PA release under stimulation is a marker of endothelial dysfunction. Plasminogen activator inhibitor type 1 (PAI-1) is the main regulatory mechanism, acting by rapid and specific inhibition of active t-PA. Increased PAI-1 levels were identified in patients with established cardiovascular disease or only with cardiovascular risk factors, such as hypertension, diabetes mellitus, dyslipidemia and obesity, and reflect the presence of endothelial dysfunction [27,28]. Sartori et al. found that hemophiliacs have impaired t-PA release after stimulation [17]. t-PA levels were significantly lower than that of age-matched healthy controls (*p* = 0.007), irrespective of the severity of hemophilia. Moreover, one in three hemophiliacs had elevated baseline PAI-1 levels. One in two hemophiliacs with mild disease and one in three hemophiliacs with moderate–severe disease had abnormal PAI-1 levels after stimulation.

One of the contributors to the onset and progression of atherosclerosis is chronic low-grade inflammation of vessel walls [29]. A small pilot study on 26 patients with hemophilia of both types and all severities investigated five biomarkers of endothelial dysfunction, namely circulating level of interleukin-6 (IL-6), tumor necrosis factor α (TNF-α), platelet selectin (P-selectin), soluble intercellular adhesion molecule-1 (sICAM-1) and monocyte chemoattractant protein-1 (MCP-1) [26]. While sICAM-1 and IL-6 were significantly higher in hemophiliacs than healthy controls, the other three biomarkers were only slightly increased, and the difference between groups did not reach statistical significance. Therefore, it is reasonable to state that hemophiliacs have at least a similar degree of endothelial dysfunction compared to the general population and can develop the same amount of atherosclerotic lesions.

The presence of low-grade systemic inflammation, identified in the previous study by increased levels of IL-6, was recently confirmed by Toenges et al. who reported increased levels of interleukins and acute phase reactants in hemophiliacs [30]. HA patients without any identifiable underlying systemic inflammatory condition, including hemophilic arthropathy, had higher levels of IL-7, IL-10, IL-12, and ferritin compared to healthy controls.

The role of microRNAs (miRNAs) in bleeding disorders and thrombosis was recently described [31]. Moreover, there is growing evidence that miRNAs participate in almost all molecular pathways of atherosclerosis [32]. miRNAs are small non-coding RNAs that regulate gene expression and many signaling pathways, including inflammation, endothelial dysfunction, platelet activation and atherosclerotic plaque formation. Studies in the general population found that increased/decreased levels of specific miRNAs are strongly associated with the progression of atherosclerosis. Recent data showed that miRNA-1, miRNA-155 and miRNA-197 were equally overexpressed in hemophiliacs, and patients without coagulopathy and documented CAD, and their levels were significantly higher in each group compared to healthy controls (*p* < 0.05) [33]. Moreover, miRNA-1, miRNA-126, miRNA-155 and miRNA-197 significantly correlated with markers of inflammation and endothelial dysfunction in hemophiliacs [33]. Network analysis established that these four miRNAs intervene in gene expression and synthesis of proteins associated with endothelial dysfunction, inflammation and atherosclerosis, confirming that hemophiliacs have enough underlying conditions to allow the development of advanced atherosclerosis [33].

### 3.3. Clinically Overt Peripheral Atherosclerotic Artery Disease

There is little experience regarding the management of PAD in hemophiliacs. A 52-year-old patient with severe HA and traditional cardiovascular risk factors—smoking, obesity and hypercholesterolemia—had atherosclerotic occlusion of the left carotid artery. He associated three-vessel disease with occlusion of the right coronary artery [34]. Therapeutic intervention consisted of initiation of low-dose aspirin along with continuation of prophylactic replacement of deficient coagulation factor.

There are few case reports of carotid angioplasty/endarterectomy in hemophiliacs [35,36,37]. A 54-year-old patient with moderate HA, hypertensive and smoker had an acute ischemic stroke as a complication of a near occlusion of the right proximal internal carotid artery [37]. A two-step treatment was implemented: emergency balloon angioplasty followed by endarterectomy due to significant residual stenosis. An ulcerated atherosclerotic carotid plaque was identified during surgery. In the long term, the patient was prescribed a statin and low-dose aspirin, while maintaining FVIII prophylaxis. A 62-year-old patient with HB and a history of stroke underwent a conventional right carotid endarterectomy for >70% stenosis of the internal carotid artery [35]. In a 73-year-old patient with mild HA, hypertensive and with a previous coronary artery bypass graft, a right carotid endarterectomy for a 70–90% stenosis was performed, followed by long-term treatment with clopidogrel [36]. A right carotid endarterectomy for a >90% stenosis was performed in a 64-year-old patient with moderate HB, recent stroke and a cluster of cardiovascular risk factors—hypertension, diabetes mellitus and smoking [36]. Perioperative coverage with deficient coagulation factor was used each time. Occurrence of postsurgical hemophilic pseudotumor and reintervention procedures were reported in two cases [35,36].

Invasive procedures for lower extremity artery disease (LEAD) are scarce. A femoral-popliteal bypass with autogenous saphenous vein graft was performed on a 61-year-old patient with mild HA for LEAD Fontaine stage IIb [38]. Replacement factor therapy was used periprocedurally.

As hemophiliacs can develop advanced atherosclerosis of carotid and lower limb arteries, they may require long-term antithrombotic treatment and revascularization procedures, both with increased hemorrhagic risk given congenital coagulopathy. Still, as long as coagulation factor concentrates or other hemostatic therapies maintain adequate hemostasis, medical and surgical treatment of cardiovascular disease in hemophiliacs parallels that of non-hemophiliacs [39]. Periprocedural management is the most challenging due to the high bleeding risk. It is recommended that hemophiliacs undergoing surgical treatment for cardiovascular diseases maintain coagulation factor levels in the range of 80–100% of normal activity from immediately before the procedure to the early postoperative period (at least until day 3 postoperatively) and at 50% until day 10–14 postoperatively [39,40,41]. Replacement therapy may be administered as an infusion or boluses [35,36,37,38].

Due to very little evidence available, an interventional approach cannot be recommended over a surgical one, neither for carotid stenosis nor for LEAD [39,42]. However, considering the durability of the stent and low rates of restenosis and complications, stenting can be considered in hemophiliacs with isolated iliac disease [42].

Current guidelines recommend antiplatelet treatment in most patients with PAD in the general population [10]. Long-term single antiplatelet therapy (SAPT) in the form of low-dose aspirin (75–100 mg/day) or clopidogrel 75 mg/day in aspirin intolerants is recommended in patients with symptomatic carotid artery stenosis, asymptomatic > 50% carotid artery stenosis, surgically revascularized or beyond 1 month after stenting [10,43]. Patients with LEAD should receive SAPT, preferably clopidogrel, only if symptomatic or have undergone revascularization [10,43,44,45]. Dual antiplatelet therapy (DAPT) in the form of low-dose aspirin and clopidogrel is reserved for patients with percutaneous revascularization for carotid stenosis or LEAD, at least in the first month after the procedure [10,45,46].

**Figure 1:**
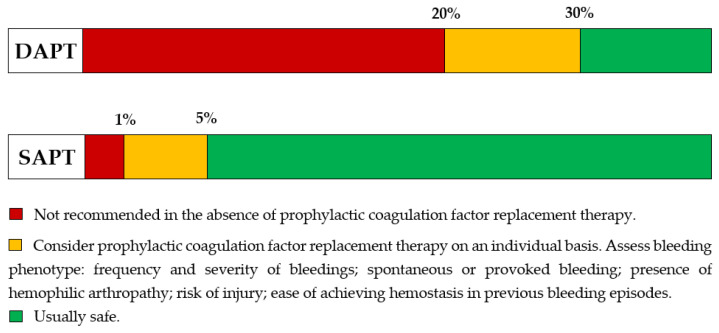
Recommendation for antiplatelet therapy depending on coagulation factor activity present in blood in hemophiliacs without inhibitors.

In hemophiliacs, due to chronic deficiency of coagulation factor, antithrombotic therapy must be modulated according to the severity of hemophilia [47]. Low-dose aspirin is safe in patients with mild hemophilia. In hemophiliacs with severe disease, treatment with low-dose aspirin requires prophylactic replacement therapy of the deficient coagulation factor. In patients with mild hemophilia, low-dose aspirin should be given on an individual basis, considering bleeding phenotype (Figure 1). Level of coagulation factor required for safe SAPT is ≥5%, while for DAPT is similar to that of long-term oral anticoagulation [48]. DAPT should not be given at coagulation factor levels < 20% without proper prophylactic replacement therapy. In patients with levels 20–30%, a recommendation should be based on an individual approach [47]. The threshold for safe anticoagulation is 30%. Therefore, many hemophiliacs will require prophylactic coagulation factor replacement therapy during DAPT. In patients with inhibitors, a decision will be made on a case-by-case basis, considering that they have a much less predictable hemostatic response to bypassing agents and therefore an increased risk of uncontrolled bleeding [48].

## 4. Discussion

Atherosclerosis is a systemic disease. PAD is defined as the presence of atherosclerotic plaques in the following territories: extracranial carotid and vertebral, mesenteric, renal, upper and lower extremities arteries. Each vascular territory with atherosclerotic lesions indicates that there is an increased risk of future fatal and non-fatal cardiovascular events. As the European Society of Cardiology Guidelines for the Diagnosis and Treatment of Peripheral Arterial Diseases points out, atherosclerosis in a vascular territory can coexist with lesions in other vascular territories and multisite artery disease is associated with the worst clinical outcomes [10]. Systematic screening for asymptomatic multisite artery disease is not indicated for any type of PAD as it would not consistently change treatment. However, further investigation may be planned in case of clinical suspicion of other localizations of PAD and/or CAD [10]. In a limited subset of patients—those scheduled for coronary artery bypass grafting—identifying severe carotid artery stenosis or advanced LEAD may affect patient management.

While several studies found that patients with hemophilia have some protection against the development of atherosclerosis [14,15,20], there is growing evidence that they are not protected either from the occurrence of atherosclerosis [16,17,18,19] or acute vascular events, the latter associated [49,50] or independent [51] of deficient coagulation factor replacement therapy. Although several hypotheses were formulated, the most important being related to the method of quantification—IMT or prevalence of atheroma plaques/degree of vascular stenosis—these differences between study results do not have a clear explanation yet [19].

Traditional cardiovascular risk factors—age, hypertension, diabetes, obesity, dyslipidemia, and smoking—appear to neutralize the potential effect that coagulation defect might exert [52]. Moreover, hemophiliacs with moderate, and especially those with severe disease, receive long-term replacement therapy that transforms the disease into a mild one [16]. Therefore, the presumed advantage of hypocoagulability fades over time. Modern treatment of hemophilia is based on extended half-life recombinant FVIII/IX concentrates, non-replacement therapies that rebalance the coagulation system by enhancing coagulation or inhibiting anticoagulant pathways, and gene therapy [53,54]. A new generation of hemophiliacs is emerging, those who receive prophylaxis with safe therapeutic agents from a young age. They will live without human immunodeficiency virus (HIV) and hepatitis C virus (HCV), but challenges of diseases associated with aging such as cardiovascular disease will remain. Given that these modern therapies have been implemented progressively over the past three decades, it is still too soon to know if the evolution of atherosclerosis will significantly change. Moreover, there are no studies to show whether increased use of prophylaxis in elderly hemophiliacs will lead to an increase in cardiovascular mortality.

Coronary artery calcification (CAC) assessed by low-dose computed tomography identified a significant atherosclerotic burden in elderly hemophiliacs, similar to that of the general population. Severe calcifications of coronary arteries were found in 24% of hemophiliacs with moderate or severe disease, aged 66.5 ± 4.6 years [55]. Younger patients develop atherosclerosis as well. In hemophiliacs with a median age of 52 years and disease of both types and all severities, the extent of coronary atherosclerosis was within normal age-dependent reference values, and independent of hemophilia type or severity [19]. As much as 14% of hemophiliacs had an increased CAC score.

Atherosclerotic carotid and femoral stenoses are not uncommon in hemophiliacs. Stenoses with a wide range of severity were identified, reaching up to vascular obstruction > 70% [15]. Severe carotid artery stenoses, from >70% to occlusion and symptomatic by stroke or transient cerebral ischemic events were reported in several patients [34,35,36,37]. LEAD is reported less frequently in hemophiliacs than CAD or stroke. Among 200 North Americans with HA, there were 49 vascular events, of which only 4 were LEAD, and in all cases, the search for LEAD was triggered by an acute coronary or cerebrovascular event [5]. In a cohort of 1054 hemophiliacs, there were 29 cases of CAD, 26 of ischemic stroke, and only 5 cases of LEAD [56]. CAC and IMT scores were higher in hemophiliacs with prior major cardiovascular events compared to those without one (*p* = 0.03) [19]. In hemophiliacs without prior major cardiovascular events, CAC and IMT scores positively correlate with population-adjusted Systematic Coronary Risk Evaluation (SCORE) value [57].

In the general population, LEAD increases 2–4 times the risk of major cardiovascular events and six times the risk of cardiovascular death [42]. Even asymptomatic patients with reduced ankle-brachial index (ABI) are at increased risk for major cardiovascular events—myocardial infarction, stroke, and cardiovascular death [10]. Claudication, the most characteristic clinical manifestation, can sometimes be a late symptom in hemophiliacs. They have exercise-limiting comorbidities such as severe hemophilic arthropathy and obesity and usually walk short distances. Therefore, they will reach the threshold of claudication only when arterial disease is in an advanced stage. Analgesics used to relieve pain from hemophilic arthropathy can mask claudication. Although possible, claudication is rarely confused with joint pain. In general, patients with established hemophilic arthropathy are familiar with joint pain and easily notice a change in its character.

Several studies reported that hemophiliacs can reach the same level of atherosclerosis as the general population [16,17,18,19]. Therefore, it is reasonable to assume that the effect of traditional cardiovascular risk factors overpowers that of decreased coagulability and promotes the onset, progression and extent of atherosclerotic lesions [19]. This is strengthened by the observation that under statin treatment, plaque structure modifies, its progression is stopped and regression may even occur [52,58,59]. A possible explanation originates in the interaction between FVIII and activated protein C (APC). APC is involved in coagulation, having a proteolytic effect on FV and FVIII, but it also exerts anti-inflammatory, antiapoptotic and cytoprotective activity on the endothelium. FVIII deficiency allows an increase in its coagulation-independent cellular effects. This mechanism is absent for FIX [60].

Endothelial dysfunction, a well-established contributor to atherosclerosis, is present in hemophiliacs. Clustering of traditional cardiovascular risk factors mainly explains altered endothelial function [17]. In hemophiliacs, other mechanisms may also contribute, such as low-grade chronic inflammation [29]. This mechanism may be implicated in patients with viral infections—HIV and HCV—and in those with hemophilic arthropathy [17,30]. In hemophiliacs with prolonged replacement therapy, protein load accumulated from coagulation factor concentrates may elicit an inflammatory response. However, there was no convincing evidence so far [25,26]. One study reported that the administration of recombinant coagulation factor concentrates did not trigger an acute inflammatory response or endothelial cell activation in HA patients with severe disease [61]. Moreover, administration of FVIII or FIX concentrate did not influence the occurrence of endothelial dysfunction, regardless of the type of product—recombinant or plasma-derived—and treatment modality—on-demand or on prophylaxis [17].

Although hemophiliacs have a burden of traditional cardiovascular risk factors and develop atherosclerosis similar to the general population, cardiovascular mortality is lower [62]. It was hypothesized that this difference resulted from coagulation factor deficiency. Firstly, the formation of occlusive thrombi may be reduced due to the generation of a smaller amount of thrombin at the point of plaque rupture [57]. Secondly, the phenotype of atherosclerotic plaques in hemophiliacs could be different from that of non-hemophiliacs, one being more stable [63]. Therefore, not only plaque load is a major determinant of cardiovascular risk, but also plaque phenotype. This hypothesis is supported by the results of mouse model studies with dabigatran etexilate, a direct thrombin inhibitor [64,65]. Apolipoprotein E-deficient mice treated with dabigatran etexilate developed atherosclerotic plaques with increased collagen and elastin content, thicker fibrous caps and a reduced number of internal elastic lamina ruptures, all these changes being stability markers of atheroma plaque [64]. Moreover, dabigatran etexilate was associated with reduced atherosclerotic plaque progression [65].

Other studies also confirm that lower thrombin activity results in less atherosclerotic lesion formation and promotes plaque stability [66,67]. Khallou-Laschet J et al. showed on an apolipoprotein E and FVIII double-deficient murine model that contribution of the intrinsic coagulation pathway, which is FVIII-dependent, has the greatest importance in the early stage of atherogenesis—stage of fatty streak—and fades over time [68]. Double-deficient mice had a more stable lesion composition compared to apolipoprotein E-deficient mice. Traces of fibrin/fibrinogen deposition, few macrophages, almost undetectable expression of vascular cell adhesion molecule-1 (VCAM-1), and absent proteoglycan and collagen deposition and platelet adhesion characterize lesions of double-deficient mice. On the contrary, apolipoprotein E-deficient mice lesions had abundant fibrin/fibrinogen deposition, high VCAM-1 expression, numerous macrophages and adherent platelets.

Atherosclerotic plaque composition in hemophiliacs was recently assessed by magnetic resonance imaging [69]. The hemophilia group consisted of 20 patients with severe disease, on prophylactic replacement treatment, with a mean age of 61 years. The control group had 20 participants, matched with cases for sex, age, presence of cardiovascular diseases and diabetes mellitus, and use of antihypertensive drugs. Plaque burden is one of the determinants of cardiovascular risk [70]. There were 11 atherosclerotic plaques in hemophiliacs and 7 in controls. Bilateral carotid plaques were identified in three hemophiliacs and one control. Although there was a difference favoring cases, statistical significance was not reached (*p* = 0.774). Another determinant of cardiovascular risk is plaque morphology which includes the presence of intraplaque hemorrhage, large lipid-rich/necrotic core and thin/ruptured fibrous cap [70]. No intraplaque hemorrhage was identified either in cases or in controls. However, three hemophiliacs had plaques with lipid-rich/necrotic core, and none of the controls. The cap was fibrous in two cases, and uncharacterizable in the third case.

Our narrative review has some limitations. Although hemophilia is the most common inherited bleeding disorder, it has a low prevalence worldwide, and most studies included small numbers of patients. Even more, it is unlikely that randomized clinical trials focusing on therapeutic interventions for PAD will ever be conducted. Some studies on subclinical atherosclerosis included both hemophilia patients and patients with von Willebrand disease to increase the number of cases with hypocoagulability, which made it much more difficult to extract necessary information. Analyzed results come from studies that enrolled patients with a mean/median age between 39.5 and 58.2 years. An older population of hemophiliacs may have more atherosclerosis. Moreover, there is heterogeneity between studies on the prevalence of risk factors for atherosclerosis and treatment with clotting factors, both with the potential to influence the onset and progression of atherosclerosis. Finally, yet importantly, one must admit that there are technical difficulties related to the measurement of IMT.

## 5. Conclusions

Hemophiliacs can reach the same degree of atherosclerotic burden as the general population. Because risk factors for the development of PAD are similar to those for CAD, hemophiliacs should undergo screening for traditional cardiovascular risk factors and PAD like the general population. Moreover, treatment of cardiovascular disease risk factors should be integrated into standard hemophilia care. Statin treatment does not raise major concerns in hemophiliacs. There is evidence that invasive treatments and long-term antithrombotic therapy are generally safe, provided that coagulation factor levels are taken into account and replacement therapy is given when necessary.

## Data Availability

Data sharing is not applicable to this article.

## References

[1] Berntorp E., Shapiro A.D. (2012). Modern haemophilia care. Lancet.

[2] White G.C., Rosendaal F., Aledort L.M., Lusher J.M., Rothschild C., Ingerslev J., Factor V., Factor I.X.S. (2001). Definitions in hemophilia. Recommendation of the scientific subcommittee on factor VIII and factor IX of the scientific and standardization committee of the International Society on Thrombosis and Haemostasis. Thromb. Haemost..

[3] Tuinenburg A., Mauser-Bunschoten E.P., Verhaar M.C., Biesma D.H., Schutgens R.E. (2009). Cardiovascular disease in patients with hemophilia. J. Thromb. Haemost..

[4] Wilding J., Zourikian N., Di Minno M., Khair K., Marquardt N., Benson G., Ozelo M., Hermans C. (2018). Obesity in the global haemophilia population: Prevalence, implications and expert opinions for weight management. Obes. Rev..

[5] Sharathkumar A.A., Soucie J.M., Trawinski B., Greist A., Shapiro A.D. (2011). Prevalence and risk factors of cardiovascular disease (CVD) events among patients with haemophilia: Experience of a single haemophilia treatment centre in the United States (US). Haemophilia.

[6] Holme P.A., Combescure C., Tait R.C., Berntorp E., Rauchensteiner S., de Moerloose P., Group A.W. (2016). Hypertension, haematuria and renal functioning in haemophilia—A cross-sectional study in Europe. Haemophilia.

[7] Badescu M.C., Badulescu O.V., Butnariu L.I., Bararu Bojan I., Vladeanu M.C., Dima N., Vlad C.E., Foia L.G., Ciocoiu M., Rezus C. (2022). Cardiovascular Risk Factors in Patients with Congenital Hemophilia: A Focus on Hypertension. Diagnostics.

[8] Biere-Rafi S., Zwiers M., Peters M., van der Meer J., Rosendaal F.R., Buller H.R., Kamphuisen P.W. (2010). The effect of haemophilia and von Willebrand disease on arterial thrombosis: A systematic review. Neth. J. Med..

[9] Kulkarni R., Soucie J.M., Evatt B.L., the Hemophilia Surveillance System Project Investigators (2005). Prevalence and risk factors for heart disease among males with hemophilia. Am. J. Hematol..

[10] Aboyans V., Ricco J.B., Bartelink M.E.L., Bjorck M., Brodmann M., Cohnert T., Collet J.P., Czerny M., De Carlo M., Debus S. (2018). 2017 ESC Guidelines on the Diagnosis and Treatment of Peripheral Arterial Diseases, in collaboration with the European Society for Vascular Surgery (ESVS): Document covering atherosclerotic disease of extracranial carotid and vertebral, mesenteric, renal, upper and lower extremity arteries Endorsed by: The European Stroke Organization (ESO)The Task Force for the Diagnosis and Treatment of Peripheral Arterial Diseases of the European Society of Cardiology (ESC) and of the European Society for Vascular Surgery (ESVS). Eur. Heart J..

[11] Visseren F.L.J., Mach F., Smulders Y.M., Carballo D., Koskinas K.C., Back M., Benetos A., Biffi A., Boavida J.M., Capodanno D. (2021). 2021 ESC Guidelines on cardiovascular disease prevention in clinical practice. Eur. Heart J..

[12] Den Ruijter H.M., Peters S.A., Anderson T.J., Britton A.R., Dekker J.M., Eijkemans M.J., Engstrom G., Evans G.W., de Graaf J., Grobbee D.E. (2012). Common carotid intima-media thickness measurements in cardiovascular risk prediction: A meta-analysis. JAMA.

[13] Kaul S., Alladi S., Mridula R.K., Bandaru S.V., Boddu D.B., Anjanikumar D., Umamashesh M. (2015). Prevalence and risk factors of carotid intima-media thickness in asymptomatic individual subjects in a tertiary care center in India. Ann. Indian Acad. Neurol..

[14] Bilora F., Dei Rossi C., Girolami B., Casonato A., Zanon E., Bertomoro A., Girolami A. (1999). Do hemophilia A and von Willebrand disease protect against carotid atherosclerosis? A comparative study between coagulopathics and normal subjects by means of carotid echo-color Doppler scan. Clin. Appl. Thromb. Hemost..

[15] Bilora F., Zanon E., Petrobelli F., Cavraro M., Prandoni P., Pagnan A., Girolami A. (2006). Does hemophilia protect against atherosclerosis? A case-control study. Clin. Appl. Thromb. Hemost..

[16] Sramek A., Reiber J.H., Gerrits W.B., Rosendaal F.R. (2001). Decreased coagulability has no clinically relevant effect on atherogenesis: Observations in individuals with a hereditary bleeding tendency. Circulation.

[17] Sartori M.T., Bilora F., Zanon E., Varvarikis C., Saggiorato G., Campagnolo E., Pagnan A., Cella G. (2008). Endothelial dysfunction in haemophilia patients. Haemophilia.

[18] Biere-Rafi S., Tuinenburg A., Haak B.W., Peters M., Huijgen R., De Groot E., Verhamme P., Peerlinck K., Visseren F.L., Kruip M.J. (2012). Factor VIII deficiency does not protect against atherosclerosis. J. Thromb. Haemost..

[19] Zwiers M., Lefrandt J.D., Mulder D.J., Smit A.J., Gans R.O., Vliegenthart R., Brands-Nijenhuis A.V., Kluin-Nelemans J.C., Meijer K. (2012). Coronary artery calcification score and carotid intima-media thickness in patients with hemophilia. J. Thromb. Haemost..

[20] Bilora F., Boccioletti V., Zanon E., Petrobelli F., Girolami A. (2001). Hemophilia A, von Willebrand disease, and atherosclerosis of abdominal aorta and leg arteries: Factor VIII and von Willebrand factor defects appear to protect abdominal aorta and leg arteries from atherosclerosis. Clin. Appl. Thromb. Hemost..

[21] Matsuzawa Y., Kwon T.G., Lennon R.J., Lerman L.O., Lerman A. (2015). Prognostic Value of Flow-Mediated Vasodilation in Brachial Artery and Fingertip Artery for Cardiovascular Events: A Systematic Review and Meta-Analysis. J. Am. Heart Assoc..

[22] Akamatsu D., Sato A., Goto H., Watanabe T., Hashimoto M., Shimizu T., Sugawara H., Sato H., Nakano Y., Miura T. (2010). Nitroglycerin-mediated vasodilatation of the brachial artery may predict long-term cardiovascular events irrespective of the presence of atherosclerotic disease. J. Atheroscler. Thromb..

[23] Mucka S., Miodonska M., Jakubiak G.K., Starzak M., Cieslar G., Stanek A. (2022). Endothelial Function Assessment by Flow-Mediated Dilation Method: A Valuable Tool in the Evaluation of the Cardiovascular System. Int. J. Environ. Res. Public Health.

[24] Flammer A.J., Anderson T., Celermajer D.S., Creager M.A., Deanfield J., Ganz P., Hamburg N.M., Luscher T.F., Shechter M., Taddei S. (2012). The assessment of endothelial function: From research into clinical practice. Circulation.

[25] Sun H., Yang M., Fung M., Chan S., Jawi M., Anderson T., Poon M.C., Jackson S. (2017). Adult males with haemophilia have a different macrovascular and microvascular endothelial function profile compared with healthy controls. Haemophilia.

[26] Bohmert S., Schubert R., Fichtlscherer S., Alesci S., Miesbach W. (2019). Endothelial Function in Patients with Severe and Moderate Haemophilia A and B. Hamostaseologie.

[27] Sillen M., Declerck P.J. (2021). A Narrative Review on Plasminogen Activator Inhibitor-1 and Its (Patho)Physiological Role: To Target or Not to Target?. Int. J. Mol. Sci..

[28] Badran M., Gozal D. (2022). PAI-1: A Major Player in the Vascular Dysfunction in Obstructive Sleep Apnea?. Int. J. Mol. Sci..

[29] Soehnlein O., Libby P. (2021). Targeting inflammation in atherosclerosis—From experimental insights to the clinic. Nat. Rev. Drug. Discov..

[30] Toenges R., Wittenbrink A., Miesbach W. (2021). Biomarkers and immunological parameters in haemophilia and rheumatoid arthritis patients: A comparative multiplexing laboratory study. Haemophilia.

[31] Jankowska K.I., Sauna Z.E., Atreya C.D. (2020). Role of microRNAs in Hemophilia and Thrombosis in Humans. Int. J. Mol. Sci..

[32] Siasos G., Bletsa E., Stampouloglou P.K., Oikonomou E., Tsigkou V., Paschou S.A., Vlasis K., Marinos G., Vavuranakis M., Stefanadis C. (2020). MicroRNAs in cardiovascular disease. Hellenic. J. Cardiol..

[33] Noone S., Schubert R., Fichtlscherer S., Hilberg T., Alesci S., Miesbach W., Klophaus N., Wehmeier U.F. (2023). Endothelial dysfunction and atherosclerosis related miRNA-expression in patients with haemophilia. Haemophilia.

[34] Zimmermann R., Staritz P., Huth-Kuhne A. (2014). Challenges in treating elderly patients with haemophilia: A focus on cardiology. Thromb. Res..

[35] Malam Y., Tsui J., Sheikh S.E., Tuddenham E.G., Baker D.M. (2012). Journal rubric. Haemophilic pseudotumour of the carotid artery. Vasc Med.

[36] Bowles L. (2012). Carotid endarterectomy in 2 patients with hemophilia. Haemophilia.

[37] Yoon C.W., Park H.K., Rha J.H. (2020). A case report and experience of endovascular treatment for a patient with hemophilia who had a hyperacute ischemic stroke. J. Stroke Cerebrovasc. Dis..

[38] Gerhardt A., Grotemeyer D., Sandmann W., Scharf R.E., Zotz R.B. (2005). A hemophilia patient with C1 domain Arg2150His mutation developed a high titer inhibitor not inhibiting autologous Factor VIII after switching to third generation recombinant product. Blood.

[39] Ferraris V.A., Boral L.I., Cohen A.J., Smyth S.S., White G.C. (2015). Consensus review of the treatment of cardiovascular disease in people with hemophilia A and B. Cardiol. Rev..

[40] Theodoropoulos K.C., Vakalopoulou S., Oikonomou M., Stavropoulos G., Ziakas A., Kanonidis I., Kassimis G. (2021). How to Manage a Patient with Haemophilia and ACS Requiring PCI: A Battle between Bleeding and Thrombosis. Medicina.

[41] Staritz P., de Moerloose P., Schutgens R., Dolan G., Group A.W. (2013). Applicability of the European Society of Cardiology guidelines on management of acute coronary syndromes to people with haemophilia—An assessment by the ADVANCE Working Group. Haemophilia.

[42] Fogarty P.F., Olin J.W., Kessler C.M., Konkle B.A., Aledort L.M. (2012). An algorithmic approach to peripheral artery disease in hemophilia: Extrapolation of management principles from noncoagulopathic patients. Blood Coagul. Fibrinolysis.

[43] Antithrombotic Trialists C., Baigent C., Blackwell L., Collins R., Emberson J., Godwin J., Peto R., Buring J., Hennekens C., Kearney P. (2009). Aspirin in the primary and secondary prevention of vascular disease: Collaborative meta-analysis of individual participant data from randomised trials. Lancet.

[44] Berger J.S., Krantz M.J., Kittelson J.M., Hiatt W.R. (2009). Aspirin for the prevention of cardiovascular events in patients with peripheral artery disease: A meta-analysis of randomized trials. JAMA.

[45] Bedenis R., Lethaby A., Maxwell H., Acosta S., Prins M.H. (2015). Antiplatelet agents for preventing thrombosis after peripheral arterial bypass surgery. Cochrane Database Syst. Rev..

[46] McKevitt F.M., Randall M.S., Cleveland T.J., Gaines P.A., Tan K.T., Venables G.S. (2005). The benefits of combined anti-platelet treatment in carotid artery stenting. Eur. J. Vasc. Endovasc. Surg..

[47] Schutgens R.E., Tuinenburg A., Fischer K., Mauser-Bunschoten E.P. (2013). Anticoagulation therapy in haemophilia. Managing the unknown. Hamostaseologie.

[48] Martin K., Key N.S. (2016). How I treat patients with inherited bleeding disorders who need anticoagulant therapy. Blood.

[49] Girolami A., Randi M.L., Ruzzon E., Zanon E., Girolami B. (2005). Myocardial infarction, other arterial thrombosis and invasive coronary procedures, in hemaophilia B: A critical evaluation of reported cases. J. Thromb. Thrombolysis.

[50] Girolami A., Ruzzon E., Fabris F., Varvarikis C., Sartori R., Girolami B. (2006). Myocardial infarction and other arterial occlusions in hemophilia a patients. A cardiological evaluation of all 42 cases reported in the literature. Acta Haematol..

[51] Fogarty P.F., Mancuso M.E., Kasthuri R., Bidlingmaier C., Chitlur M., Gomez K., Holme P.A., James P., Kruse-Jarres R., Mahlangu J. (2015). Presentation and management of acute coronary syndromes among adult persons with haemophilia: Results of an international, retrospective, 10-year survey. Haemophilia.

[52] Girolami A., Sambado L., Lombardi A.M. (2013). The impact of blood coagulability on atherosclerosis and cardiovascular disease: A rebuttal. J. Thromb. Haemost..

[53] Marchesini E., Morfini M., Valentino L. (2021). Recent Advances in the Treatment of Hemophilia: A Review. Biologics.

[54] Miesbach W., Klamroth R., Oldenburg J., Tiede A. (2022). Gene Therapy for Hemophilia-Opportunities and Risks. Dtsch. Arztebl. Int..

[55] Tuinenburg A., Rutten A., Kavousi M., Leebeek F.W., Ypma P.F., Laros-van Gorkom B.A., Nijziel M.R., Kamphuisen P.W., Mauser-Bunschoten E.P., Roosendaal G. (2012). Coronary artery calcification in hemophilia A: No evidence for a protective effect of factor VIII deficiency on atherosclerosis. Arterioscler. Thromb. Vasc. Biol..

[56] Wang J.D., Chan W.C., Fu Y.C., Tong K.M., Chang S.T., Hwang W.L., Lin C.H., Tsan Y.T. (2015). Prevalence and risk factors of atherothrombotic events among 1054 hemophilia patients: A population-based analysis. Thromb. Res..

[57] Makris M., Van Veen J.J. (2012). Reduced cardiovascular mortality in hemophilia despite normal atherosclerotic load. J. Thromb. Haemost..

[58] Nissen S.E., Nicholls S.J., Sipahi I., Libby P., Raichlen J.S., Ballantyne C.M., Davignon J., Erbel R., Fruchart J.C., Tardif J.C. (2006). Effect of very high-intensity statin therapy on regression of coronary atherosclerosis: The ASTEROID trial. JAMA.

[59] Shin E.S., Garcia-Garcia H.M., Okamura T., Serruys P.W. (2012). Effect of statins on coronary bifurcation atherosclerosis: An intravascular ultrasound virtual histology study. Int. J. Cardiovasc. Imaging.

[60] Mosnier L.O., Zlokovic B.V., Griffin J.H. (2007). The cytoprotective protein C pathway. Blood.

[61] van Bladel E.R., Tuinenburg A., Roest M., de Groot P.G., Schutgens R.E. (2014). Factor VIII concentrate infusion in patients with haemophilia results in decreased von Willebrand factor and ADAMTS-13 activity. Haemophilia.

[62] Shapiro S., Benson G., Evans G., Harrison C., Mangles S., Makris M. (2022). Cardiovascular disease in hereditary haemophilia: The challenges of longevity. Br. J. Haematol..

[63] Kamphuisen P.W., ten Cate H. (2014). Cardiovascular risk in patients with hemophilia. Blood.

[64] Kadoglou N.P., Moustardas P., Katsimpoulas M., Kapelouzou A., Kostomitsopoulos N., Schafer K., Kostakis A., Liapis C.D. (2012). The beneficial effects of a direct thrombin inhibitor, dabigatran etexilate, on the development and stability of atherosclerotic lesions in apolipoprotein E-deficient mice: Dabigatran etexilate and atherosclerosis. Cardiovasc. Drugs Ther..

[65] van Gorp R.H., Dijkgraaf I., Broker V., Bauwens M., Leenders P., Jennen D., Dweck M.R., Bucerius J., Briede J.J., van Ryn J. (2021). Off-target effects of oral anticoagulants—Vascular effects of vitamin K antagonist and non-vitamin K antagonist oral anticoagulant dabigatran etexilate. J. Thromb. Haemost..

[66] Borissoff J.I., Otten J.J., Heeneman S., Leenders P., van Oerle R., Soehnlein O., Loubele S.T., Hamulyak K., Hackeng T.M., Daemen M.J. (2013). Genetic and pharmacological modifications of thrombin formation in apolipoprotein e-deficient mice determine atherosclerosis severity and atherothrombosis onset in a neutrophil-dependent manner. PLoS ONE.

[67] Bea F., Kreuzer J., Preusch M., Schaab S., Isermann B., Rosenfeld M.E., Katus H., Blessing E. (2006). Melagatran reduces advanced atherosclerotic lesion size and may promote plaque stability in apolipoprotein E-deficient mice. Arterioscler. Thromb. Vasc. Biol..

[68] Khallou-Laschet J., Caligiuri G., Tupin E., Gaston A.T., Poirier B., Groyer E., Urbain D., Maisnier-Patin S., Sarkar R., Kaveri S.V. (2005). Role of the intrinsic coagulation pathway in atherogenesis assessed in hemophilic apolipoprotein E knockout mice. Arterioscler. Thromb. Vasc. Biol..

[69] Hop H., Potze J.H., van den Berg-Faaij S., Borra R.J.H., Zheng K.H., Nederveen A.J., Meijer K., Kamphuisen P.W. (2021). Carotid plaque composition in persons with hemophilia: An explorative study with multi-contrast MRI. Thromb. Res..

[70] Zavodni A.E., Wasserman B.A., McClelland R.L., Gomes A.S., Folsom A.R., Polak J.F., Lima J.A., Bluemke D.A. (2014). Carotid artery plaque morphology and composition in relation to incident cardiovascular events: The Multi-Ethnic Study of Atherosclerosis (MESA). Radiology.

